# Identification and analysis of long non-coding RNAs and mRNAs in chicken macrophages infected with avian infectious bronchitis coronavirus

**DOI:** 10.1186/s12864-020-07359-3

**Published:** 2021-01-20

**Authors:** Hao Li, Pengfei Cui, Xue Fu, Lan Zhang, Wenjun Yan, Yaru Zhai, Changwei Lei, Hongning Wang, Xin Yang

**Affiliations:** 1 Key Laboratory of Bio-Resources and Eco-Environment, Ministry of Education, College of Life Science, Sichuan University, Chengdu, 610064 China; 2 Animal Disease Prevention and Food Safety Key Laboratory of Sichuan Province, Chengdu, 610064 China

**Keywords:** IBV, lncRNA, HD11, Coronavirus, Chicken, Gga-miR-30d, miR-146a-5p

## Abstract

**Background:**

Avian infectious bronchitis virus (IBV) is a gamma coronavirus that severely affects the poultry industry worldwide. Long non-coding RNAs (lncRNAs), a subset of non-coding RNAs with a length of more than 200 nucleotides, have been recently recognized as pivotal factors in the pathogenesis of viral infections. However, little is known about the function of lncRNAs in host cultured cells in response to IBV infection.

**Results:**

We used next-generation high throughput sequencing to reveal the expression profiles of mRNAs and lncRNAs in IBV-infected HD11 cells. Compared with the uninfected cells, we identified 153 differentially expressed (DE) mRNAs (106 up-regulated mRNAs, 47 down-regulated mRNAs) and 181 DE lncRNAs (59 up-regulated lncRNAs, 122 down-regulated lncRNAs) in IBV-infected HD11 cells. Moreover, gene ontology (GO) and pathway enrichment analyses indicated that DE mRNAs and lncRNAs were mainly involved in cellular innate immunity, amino acid metabolism, and nucleic acid metabolism. In addition, 2640 novel chicken lncRNAs were identified, and a competing endogenous RNA (ceRNAs) network centered on gga-miR-30d and miR-146a-5p was established.

**Conclusions:**

We identified expression profiles of mRNAs and lncRNAs during IBV infection that provided new insights into the pathogenesis of IBV.

**Supplementary Information:**

The online version contains supplementary material available at 10.1186/s12864-020-07359-3.

## Background

Avian infectious bronchitis (IB) is a highly contagious viral disease of chicken caused by infectious bronchitis virus (IBV). The disease incurs huge economic losses to the poultry industry annually [1]. IBV belongs to the gamma coronaviruses family. Like other coronaviruses, IBV contains a 27.6 kb single-stranded, positive-sense RNA genome, which encodes for polyproteins 1a and 1b, four structural proteins (the spike [S], envelope [E], membrane [M], and nucleocapsid [N] proteins), and several accessory proteins (3a, 3b, 5a, and 5b) [2]. The disease is manifested by clinical–pathological signs in several tissues, including the respiratory tract, kidneys, gut, oviduct, and testes, resulting in poor performance of egg-laying birds and poor quality of meat. Moreover, the disease can be lethal in several cases [3, 4]. Vaccination is the most reliable approach to control IBV. However, existing vaccines cannot provide effective protection owing to the high frequency of mutations and recombination of the IBV genome between viruses with large genetic differences. Therefore, commercial vaccines often fail or only provide partial protection against IBVs [5], posing a major challenge to the poultry industry.

Long non-coding RNAs (lncRNAs) are transcripts longer than 200 nucleotides but without protein-coding capacity [6]. LncRNAs are critical regulators of a wide range of biological processes, including cell proliferation, differentiation, apoptosis, autophagy, tissue repair, and remodeling [7]. Several studies have demonstrated that lncRNAs function at the host–pathogen interface to regulate viral infections either by innate immune responses at several levels including activation of pathogen-recognition receptors or by epigenetic, transcriptional, and posttranscriptional effects [8]. For example, the latest research reported that lncRNA Malat1, a negative regulator of antiviral type I IFN (IFN-I) production, suppressed antiviral innate responses by targeting *TDP43* activation via the RNA–RBP interactive network [9]. Although the nature of lncRNAs is well characterized in mammals, little is known about their functions in birds, especially in the field of antivirals for poultry [10]. Furthermore, except for loc107051710, lncRNA L11530, and lncRNA L09863 [11, 12], little is known about lncRNA-mediated innate immune response in chicken. The functions of lncRNAs in anti-IBV immune response in chickens remain unclear.

LncRNAs regulate viral infections in multiple ways, such as epigenetic regulation and promotion of viral latency, protein scaffolding and nuclear localization, alternative splicing, and transcriptional regulation of mRNA via miRNA “sponges” [13]. One of the primary mechanisms of functioning of lncRNAs is by competing for shared microRNAs with mRNAs—known as the competing endogenous RNA (ceRNA) hypothesis [14]. We have previously analyzed the miRNAs of IBV-infected chicken kidney tissue and obtained two differentially expressed miRNAs, namely gga-miR-30d and miR-146a-5p, which are encoded by chicken chromosomes 1 and 13, respectively [15]. Next, an in vitro study demonstrated that gga-miR-30d inhibited IBV replication in HD11 cells by targeting *USP47* [16], whereas miR-146a-5p promoted IBV replication in HD11 cells by targeting *IRAK2* and *TNFRSF18* [17].

We further analyzed the expression patterns of lncRNAs and mRNAs in IBV-infected HD11 cells using next-generation high throughput sequencing techniques. Differential expression and co-expression network analysis were conducted to identify interactions between mRNAs and lncRNAs to understand their possible functions in IBV infection. Moreover, a ceRNA network based on gga-miR-30d and miR-146a-5p was established. These results reveal a new data platform to conduct functional studies of chicken lncRNAs and provide valuable information on new therapeutic approaches to control IBV.

## Results

### Replication status of IBV in HD11 cells

**Fig. 1:**
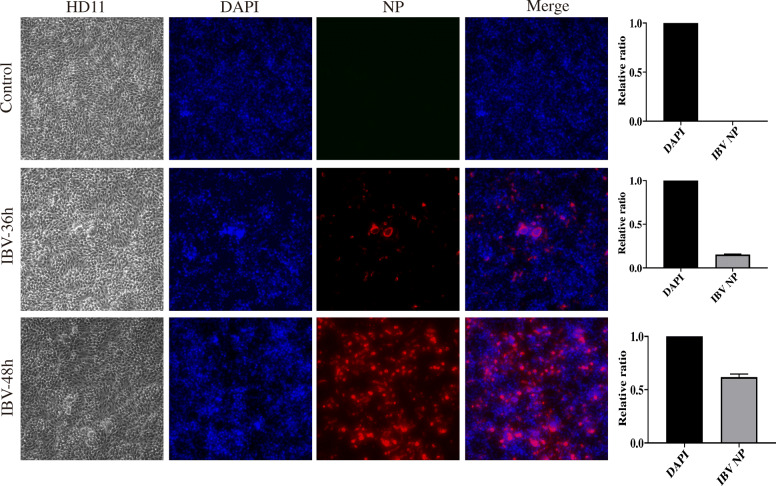
Indirect immunofluorescence assay (IFA) of HD11 cells infected by IBV. Cells were fixed at the indicated time and infected with viruses, followed by immunofluorescence (IF) staining of IBV N protein (red). Cell nuclei were visualized by DAPI staining. Merge refers to an overlap of DAPI and NP (amplification: 200×). The histogram represents the ratio of relative intensity of red fluorescence (IBV N protein) to blue fluorescence (Nucleus)

The expression of non-coding RNAs (ncRNAs) in cells is closely related to the stage of virus infection [13]. To confirm the status of IBV in HD11 cells, indirect fluorescent immunoassay (IFA) was performed after IBV infection for 0, 36, or 48 h **(**Fig. 1**)**. The infection rate of the virus was calculated using the relative ratio of red fluorescence (IBV N protein) and blue fluorescence (cell nucleus) using ImageJ [18]. The results showed that the virus replication started a little after 36 h of infection; however, it replicated vigorously after 48 h of infection (manifested by rupture and collapse of cells, cell aggregation under bright-field microscopy, and significant red fluorescence). No red fluorescence was observed in mock-infected cells. Cytopathies affect the stability of nucleic acids in the cells. Therefore, cells after 36 h of infection were selected to extract total RNA and build the libraries.

### RNA libraries establishment and lncRNA identification

Total RNA was extracted from 36 h post-infected HD11 cells (Exp 1, 2, and 3) and mock-infected cells (CK 1, 2, and 3). After high-throughput sequencing, six libraries with an average of 140,317,022 raw reads and 21,047,553,300 bases were obtained. Nucleotides with a quality value above 30 (Q30) in reads were ranged from 94.09 to 94.4%. After data filtering and quality control, an average of 127,609,381 (90.95%) clean reads with 19,141,407,250 high-quality bases were retained. Following the removal of rRNAs, clean reads were mapped to the chicken reference genome. The percentage mapping rates of six libraries ranged from 91.565 to 92.24% (Table 1).

**Table 1:** Overview of the RNA sequencing data

Sample	Raw Data Reads No.	Bases (bp)	Q30 (bp)	Clean Reads No	Clean Bases (bp)	Clean Reads %	Genome Mapping Rate	Sequencing Mode
**C-1**	**150,897,246**	**22,634,586,900**	**21,331,618,679 (94.24%)**	**137,417,676**	**20,612,651,400**	**91.06**	**125,822,533 (91.56%)**	**Paired-end,2 × 150 bp**
**C-2**	**137,054,082**	**20,558,112,300**	**19,407,891,463 (94.4%)**	**126,612,888**	**18,991,933,200**	**92.38**	**116,736,730 (92.20%)**	**Paired-end,2 × 150 bp**
**C-3**	**138,645,590**	**20,796,838,500**	**19,632,609,578 (94.4%)**	**124,766,544**	**18,714,981,600**	**89.98**	**114,421,545 (91.71%)**	**Paired-end,2 × 150 bp**
**E-1**	**129,518,036**	**19,427,705,400**	**18,289,744,390 (94.14%)**	**117,058,784**	**17,558,817,600**	**90.38**	**107,868,528 (92.15%)**	**Paired-end,2 × 150 bp**
**E-2**	**154,994,906**	**23,249,235,900**	**21,875,735,326 (94.09%)**	**139,996,898**	**20,999,534,700**	**90.32**	**129,086,227 (92.21%)**	**Paired-end,2 × 150 bp**
**E-3**	**130,792,272**	**19,618,840,800**	**18,520,223,188 (94.4%)**	**119,803,500**	**17,970,525,000**	**91.59**	**110,508,187 (92.24%)**	**Paired-end,2 × 150 bp**
**Average**	**140,317,022**	**21,047,553,300**	**19,842,970,437 (94.27%)**	**127,609,381**	**19,141,407,250**	**90.95**	**117,407,291 (92.01%)**	

Q30: nucleotides with a quality value above 30 in reads

Genome mapping rate: the percentage of reads mapped to the reference genome

**Fig. 2:**
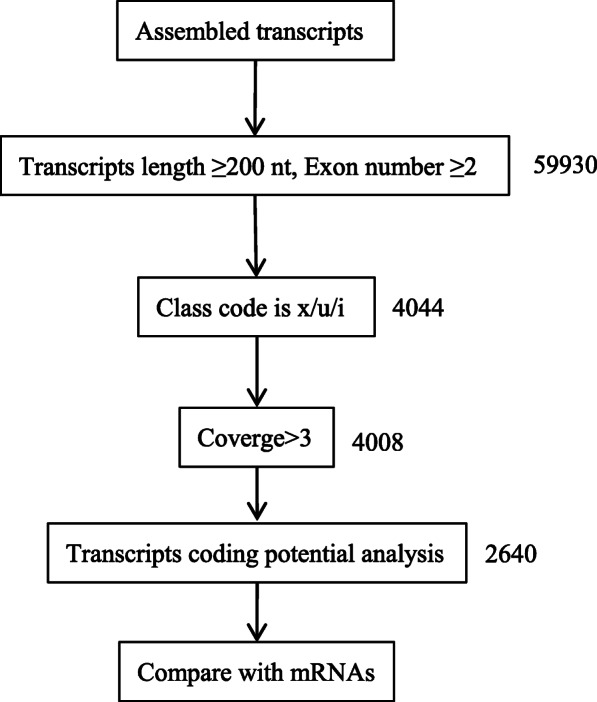
LncRNA identification Process, “u” (unknown intergenic transcript), “i” (a transfrag falling entirely within a reference intron), and “x” (exonic overlap with reference on the opposite strand). The number on the right side of the picture represents the number of transcripts filtered out from each step

The lncRNAs were identified as follows **(**Fig. 2**)**. We used StringTie (version 1.2.4) software to assemble the transcripts based on the comparison results of HISAT2 (version 2.1.0). Transcripts with uncertain strand orientation were removed. The remaining assembled transcripts for lncRNAs were screened for transcripts with length ≥ 200 nucleotides and exon number ≥ 2 to obtain 59,930 transcripts. Transcripts whose class-code was x/u/i were screened to obtain 4044 transcripts. Moreover, transcripts with cover > 3 in at least one sample were screened to obtain 4008 transcripts. We used PLEK (version 1.2), Coding-Non-Coding Index (CNCI; version 2.0), and PfamScan (version 1.6) to analyze the coding potential of candidate lncRNAs. All three software revealed that new transcripts without coding potential were high confidence lncRNAs. Ultimately, 2640 lncRNAs were identified.

**Fig. 3:**
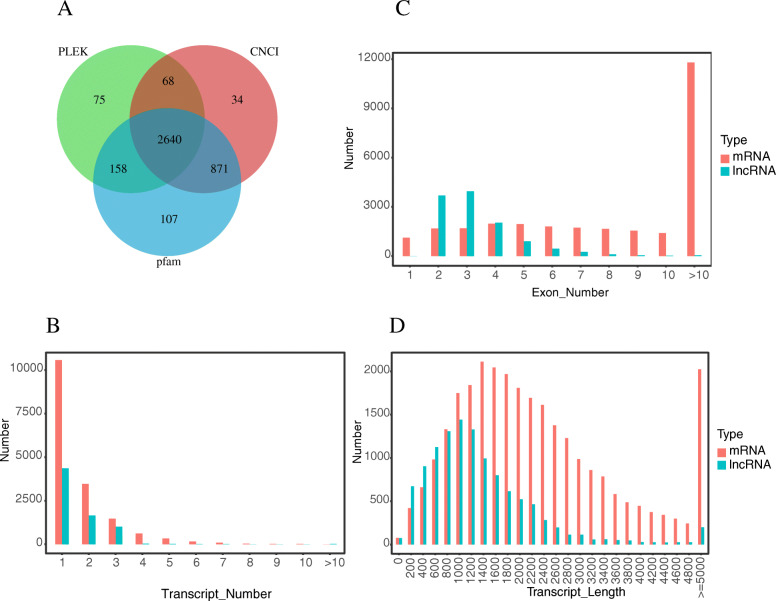
Prediction and characterization of novel lncRNAs. (A) Three different colored circles represent three different lncRNA prediction software. Overlapping areas of Venn diagrams represent the number of newly identified lncRNAs. (B) Distribution of the number of transcripts in lncRNAs and mRNAs in chicken HD11 cells. (C) Distribution of the number of exons in lncRNAs and mRNAs in chicken HD11 cells. (D) Distribution of transcript lengths in lncRNAs and mRNAs in chicken HD11 cells

To further identify the characteristics of lncRNAs, we compared predicted lncRNAs with mRNAs for transcript number, length, and exon number **(**Fig. 3**)**. The result showed that the number of mRNA transcripts was higher than that of lncRNAs. With respect to the transcript length, the predicted lncRNAs were primarily concentrated between 200 and 3000 bp, whereas mRNAs were mainly of a length between 1400 and 5000 bp. In addition, the majority of lncRNAs contained two to three exons and very few had more than 10 exons. However, the majority of mRNAs contained more than 10 exons. In summary, compared with mRNAs, lncRNAs had fewer and shorter transcripts, and fewer exons.

### Expression profiles of lncRNAs and mRNAs in IBV-infected HD11 cells

**Fig. 4:**
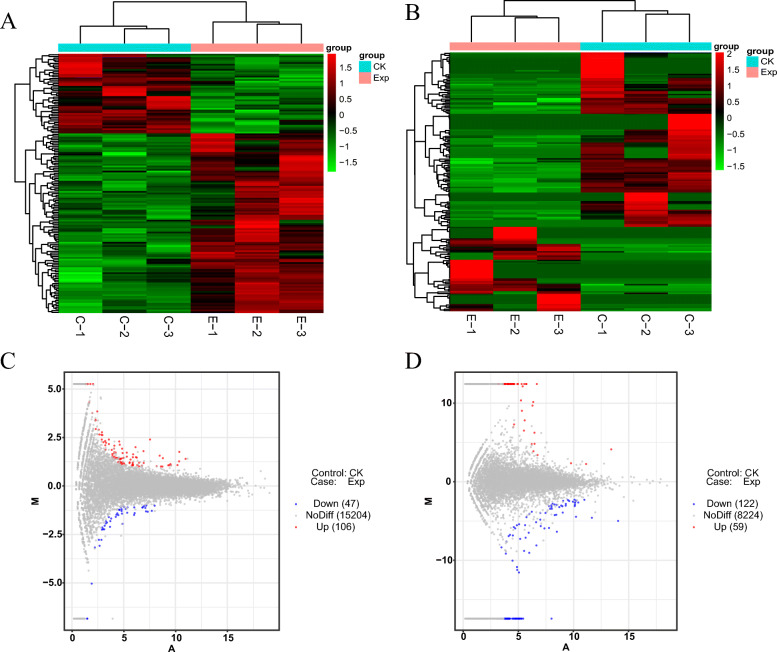
Differentially expressed genes (DEGs) and differentially expressed (DE) lncRNAs analysis. Exp (IBV infected HD11 cells) vs CK (mock infected HD11 cells) (A, C) Heat map and M-A map for mRNAs expression in control and IBV-stimulated avian HD11 cells at 36 h post-infection. (B, D) Heat map and M-A map for lncRNAs in control and IBV-stimulated avian HD11 cells at 36 h post-infection

In total, we obtained 15,358 mRNAs and 11,510 lncRNAs. Moreover, 153 mRNAs were differentially expressed, with 106 mRNAs significantly up-regulated and 47 mRNAs significantly down-regulated (Additional File 1). In addition, the expression of 181 lncRNAs changed significantly. Among these, 59 lncRNAs were up-regulated and 122 were down-regulated (Additional File 2). Heat map and M-A map enrichment analyses **(**Fig. 4**)** revealed that compared with the control group, the expression profiles of lncRNAs and mRNAs changed significantly after 36 h of IBV infection.

### LncRNA target gene prediction

**Fig. 5:**
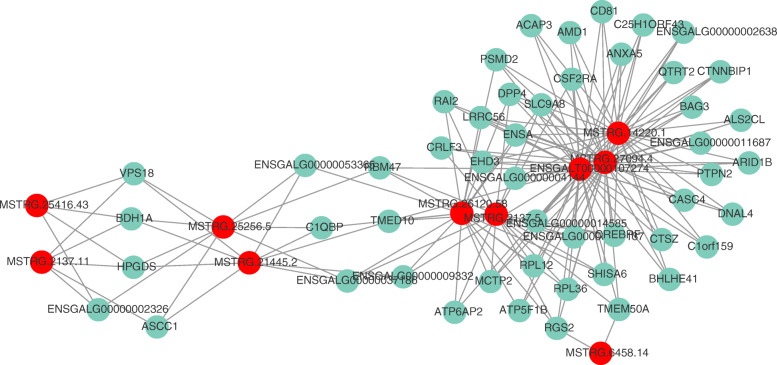
Co-expression network of DE lncRNAs and mRNAs based on Pearson’s correlation coefficient. The top 10 DE lncRNAs and their 50 most frequently altered relative mRNAs with 174 connection edges in IBV-infected HD11 cells are shown. The red node denotes lncRNA and the blue node represents genes

LncRNAs regulate genes in a cis or trans manner. We enumerated the top 10 DE lncRNAs and their possible target genes by searching the gene-encoding protein within 100 kb upstream and downstream of lncRNAs. These genes were considered potential cis-regulated target genes corresponding to the lncRNAs. For example, SHISA6, located 19,659 bp downstream of MSTRG8180, was considered a potential target gene of MSTRG8180 **(**Additional File 3**)**. To predict the trans-regulated target genes of lncRNAs, the top 10 DE lncRNAs and 50 most relevant mRNAs were selected to construct the co-expression network of lncRNA–mRNA pairs based on Pearson’s correlation coefficient by Cytoscape (Additional File 4). As shown in Fig. 5, the network contained 174 edges. The majority of lncRNAs had multiple target genes, which were related to other lncRNAs, thus forming a large and complex co-expression network.

### Pathway analysis of regulated lncRNAs and mRNAs after IBV infection in HD11

**Fig. 6:**
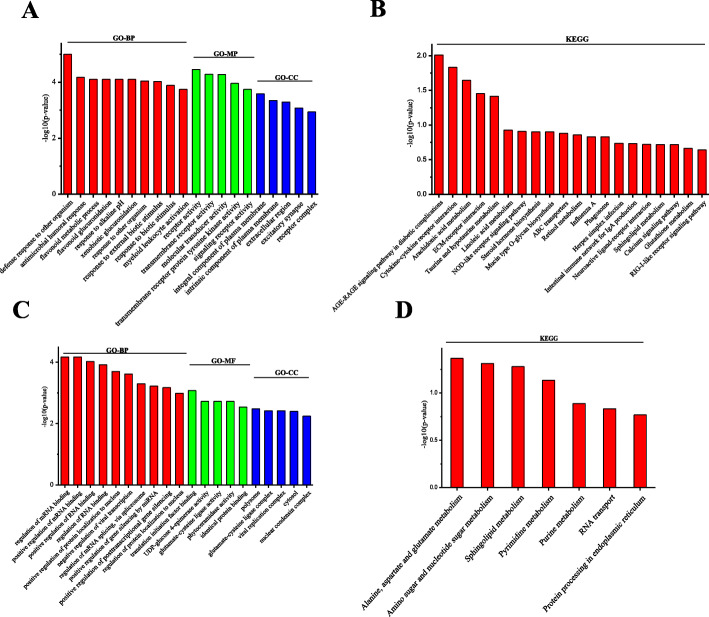
The GO and KEGG enrichment analyses of DEGs and the target genes of DE lncRNAs. (A) The top 10 GO-BP, 5 GO-MF, and 5 GO-CC terms of DEGs. (B) The top 20 KEGG terms of DEGs. (C) The top 10 GO-BP, 5 GO-MF, and 5 GO-CC terms of DE lncRNAs. (D) The KEGG terms of DE lncRNAs

To further explore the functions of these DEGs and DE-lncRNAs following IBV infection, GO categorization and pathway analyses were performed. Significantly enriched GO terms (top 10 biological processes [BP], top 5 molecular functions [MFs], and top 5 cellular components [CCs]) and KEGG terms for mRNAs and lncRNAs are listed in Fig. 6.

As shown in Fig. 6a, the GO categorization indicated that DEGs were mainly enriched in biological processes of cellular immunity in response to external stimuli. The top three enriched GO terms were related to defense response to other organisms (GO: 0098542), antimicrobial humoral response (GO: 0019730), and flavonoid metabolism (GO: 0009812). Further, GO-MF and GO-CC analyses identified cytosol and transcription factor binding, respectively. Furthermore, the KEGG pathway analysis revealed that the identified mRNAs mainly participated in protein processing in the AGE–RAGE signaling pathway in diabetic complications (ID: gga04933), cytokine–cytokine receptor interaction (ID: gga04060), and arachidonic acid metabolism (ID: gga00590). The top 20 pathways are shown in Fig. 6b.

The GO enrichment analysis of DE lncRNAs showed them to be mainly involved in the regulation of mRNA and RNA binding during IBV replication. For example, the most enriched GO–BP terms were regulation of mRNA binding (GO: 1902415), positive regulation of mRNA binding (GO: 1902416), and positive regulation of RNA binding (GO: 1905216). In addition, several terms directly related to virus infection were found, for example, negative regulation of viral transcription (GO: 0032897 top 6), viral transcription (GO: 0019083 top 11), and regulation of viral transcription (GO: 0046782 top 12). The translation initiation factor binding (GO: 0031369), UDP-glucose 4-epimerase activity (GO: 0003978), glutamate–cysteine ligase activity (GO: 0004357), polysome (GO: 0005844), and glutamate–cysteine ligase complex (GO: 0017109), and viral replication complex (GO: 0019034) were the top three enriched GO-MP and GO-CC terms, respectively (Fig. 6c). The KEGG pathway analysis indicated that lncRNA target genes mainly participated in alanine, aspartate, and glutamate metabolism (gga00250), amino sugar and nucleotide sugar metabolism (gga00520), and sphingolipid metabolism (gga00600). The enrichment pathways are listed in Fig. 6d.

### Immune-related mRNA and lncRNA analysis

Innate immunity is a crucial defense mechanism of cells against viral infections. We screened the DEGs related to innate immunity in HD11 cells infected with IBV for 36 h. These genes included *CSF2, IFIT5, IL15, IL1RAPL1, IL22, IL8, MX1, NR1H4, S100A9, SYK, TRAF5, TRIM67,* and *ZFPM2.* Among these, *IL15*, *IL1RAPL1,* and *SYK* were significantly down-regulated, whereas some anti-viral genes, such as *IFIT5* and *MX1,* and some inflammatory factor genes, such as *IL8* and *IL22,* were significantly up-regulated (Additional File 1).

**Fig. 7:**
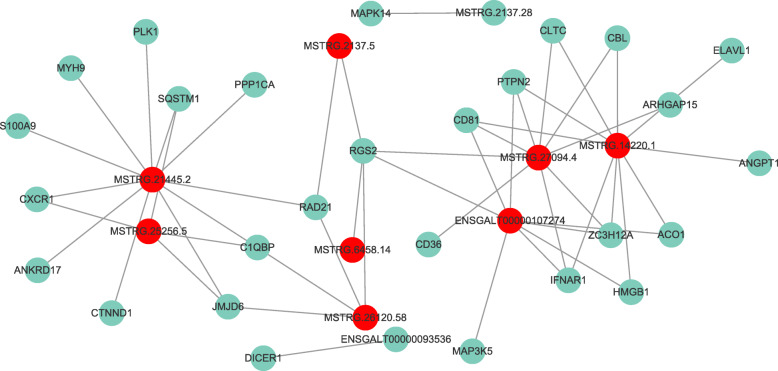
The interaction network of 10 DE lncRNAs and immune-related targets in IBV-infected chicken HD11 cells. The red node denotes lncRNA and the blue node represents immune-related gene

We next analyzed the DE-lncRNAs and screened their target genes related to immunity. We constructed a network diagram using correlation coefficients (Fig. 7**)** that revealed a complex regulatory network between lncRNAs and immune genes. One lncRNA participated in the regulation of multiple genes in different ways, and one gene was regulated by multiple lncRNAs. For example, lncRNA MSTRG.14220.1 and MSTRG.21445.2 (Additional File 5) were related to at least 10 or 11 immune genes, and they were speculated to function in immune regulation in HD11 cells.

### LncRNA–miRNA–mRNA regulation network analysis

**Fig. 8:**
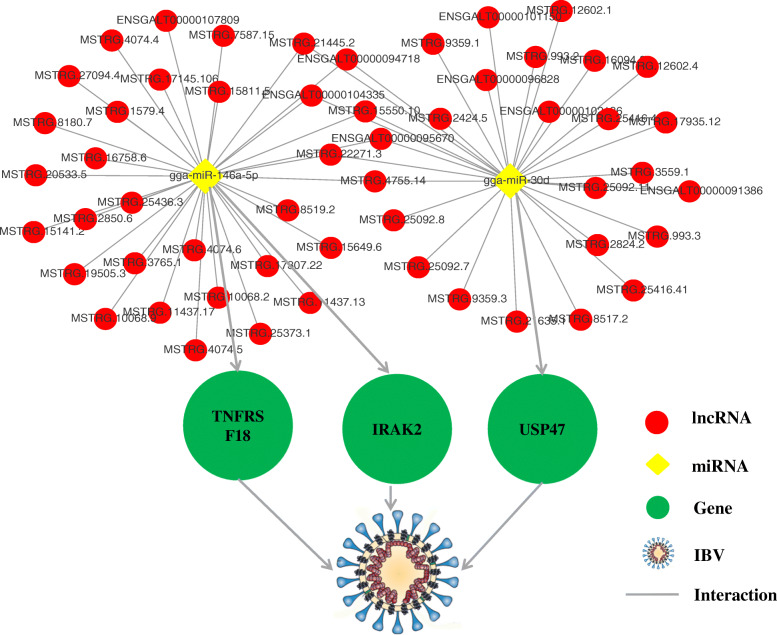
The competitive endogenous RNA (ceRNAs) network centered on gga-miR-30d and miR-146a-5p. The relationship between miRNAs and target genes has been shown [16, 17]. The expression of the lncRNAs in the picture had changed significantly in IBV-infected HD11 cells, and they were predicted to interact with miRNAs at the nucleic acid sequence level

LncRNAs can affect the gene expression through a variety of strategies [13]. We have previously reported that miR-146a-5p and gga-miR-30d had significant regulatory roles in IBV infection in HD11 cells [16, 17]. We screened for lncRNAs that interacted with miR-146a-5p and gga-miR-30d. The results revealed 1563 lncRNAs to interact with gga-miR-30d. Among these, 30 lncRNAs were differentially expressed after 36 h of IBV infection. A total of 1563 lncRNAs were found to interact with miR-146a-5p, and the expression of 32 lncRNAs changed significantly after 36 h of IBV infection. We constructed a miRNA–mRNA–lncRNA interaction network on the basis of potential interactions between them **(**Fig. 8 and Additional File 6**)**. It is believed that these lncRNAs either function alone or compete for miR-146a-5p and gga-miR-30d with three genes (*USP47, IRAK2*, and *TNFRSF18*) to regulate IBV infection. In addition, eight lncRNAs (MSTRG.8180.7, MSTRG.4755.14, MSTRG.22271.3, MSTRG.21445.2, MSTRG.15550.10, ENSGALT00000104335, ENSGALT00000095670, and ENSGALT00000094718) were found to interact with both miR-146a-5p and gga-miR-30d (Fig. 8**)**.

### RT-qPCR validation

**Fig. 9:**
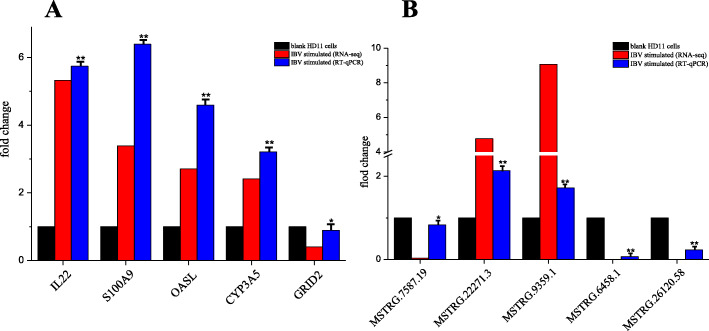
Results of qPCR analysis of selected mRNAs and lncRNAs. (A) Results of selected mRNAs. (B) Results of selected lncRNAs. All experiments were performed at least in triplicate. Significant differences between the treated and control groups are expressed as *p < 0.05 and **p < 0.01, respectively

To validate the high-throughput sequencing results, we performed qPCR to detect the expression of lncRNAs and mRNAs in HD11 cells. Five lncRNAs and five mRNAs were selected randomly for qPCR to determine their relative expression (Table 2). The results are shown in Fig. 9. The qPCR results indicated that the expression patterns of these lncRNAs and mRNAs were consistent with those by RNA-sequencing.

**Table 2:** Primers for qPCR

mRNA/lncRNA	Forword primer	Reverse primer
MSTRG.7587.19	GACCGTCGTGAGACAGGTTAGT	CTCCTCAGCCAAGCACATACAC
MSTRG.22271.3	GCAACTTCCAGAGACCACAGAA	CTCCAGCCACCAAGCACAAC
MSTRG.9359.1	GCTACCAAGCAATGTGTTCCAC	AGTGAGGCAAGTGAGGAGAAGG
MSTRG.6458.14	GGTGTGGCTGGTGGACTGTA	AGCCGCACCTGTAGTGAGAC
MSTRG.26120.58	ACGACATTAGGCGGTACGGAAT	GAGGCTGGAGTGGCACAAGA
IL22	CAGGCTCTCAGATCAGGACACT	TTCATCATGTAGCAGCGGTTGT
S100A9	TGGTGAAGTGATGCTCCTGAT	GATGTTGGTGTTGGTGTTGGT
OASL	GGTCAAGCACTGGTACAAGGAG	CAGGCGTAGATGGTCAGCAG
CYP3A5	ACAGCAGAAGGTGGTGAATGAA	GCCATGTCGAGGTATTCCAACT
GRID2	ACTCACATCACCACAACAACCT	TGCCGAAGGAGAAGCCAGAA
chGAPDH	ACTGTCAAGGCTGAGAACGG	GCTGAGGGAGCTGAGATGA

## Discussion

The fact that almost all isolated wild-type IBV strains cannot adapt to cell lines restricts their in-depth research [19]. In 2017, Han et al. reported that the IBV Beaudette strain could be serially passaged in HD11 cells and triggered cytopathic effects [20]. HD11 is an avian myelocytomatosis virus (MC29) transformed chicken macrophage-like cell line [21]. Because macrophages function in both innate and adaptive immunity [22], it is imperative to study the response of ncRNAs in macrophages to viral stimulation. In the present study, we studied the mRNA–lncRNA regulatory network following IBV infection of HD11 cells.

The development of next-generation, high-throughput sequencing techniques has enabled us to obtain global RNA expression profiling. Recently, several studies have implicated genes and ncRNAs in IBV infection. Cong et al. [23] performed a transcriptome analysis in chicken kidney tissues infected with IBV strain ck/CH/LDL/091022. Their results showed that *IL6, STAT1, MYD88, IRF1,* and *NFKB2* as key immune and inflammatory responses genes. We conducted in vitro studies to assess the changes in the expression of genes related to innate immunity in IBV-infected HD11 cells using high-throughput sequencing. The results revealed that certain inflammatory factors (*IL15, IL1RAPL1*, *IL22*, and *IL8*) and some antiviral genes such as *IFIT5* and *MX1* were differentially expressed following IBV infection. However, in addition to the above genes, the typical signaling pathways for transcriptional activation of chicken IFNs (such as Toll-like receptors), their downstream signaling pathways (such as the JAK/STAT pathway), and interferon-stimulated genes (ISGs) did not change significantly. IBV can use multiple strategies to counteract the IFN response. Certain studies report that IBV delays the activation of the IFN response and inhibits IFN-mediated phosphorylation and translocation of *STAT1* [24–26]. We did not detect changes in typical innate immune signaling pathway factors at mRNA levels during the early replication stage of IBV, which could be one of the ways by which IBV escapes the host’s innate immunity.

It is reported that certain lncRNAs regulate the expression of immune genes to regulate viral infections. For example, lncRNA NEAT1 is associated with the expression of IL-8, eventually affecting the virus infection [27]. Similarly, the lncRNA BST2/BISPR is induced by IFN and regulates the expression of the antiviral factor tetherin [28]. However, unlike coding genes, lncRNAs are poorly conserved at the level of the primary sequence [13]. Very few lncRNAs related to immunity in chickens have been studied. We studied the lncRNA-related immune response in IBV-infected HD11 cells. LncRNAs, such as lncRNA MSTRG.14220.1 (predicted target genes: *ACO1*, *ELAVL1*, *CD81*, *HMGB1*, *ZC3H12A*, *ANGPT1*, *CBL*, *IFNAR1*, *CLTC*, and *PTPN2*) and MSTRG.21445.2 (predicted target genes: *JMJD6*, *CXCR1*, *S100A9*, *SQSTM1*, *CTNND1*, *MYH9*, *ANKRD17*, *PPP1CA*, *PLK1*, *RAD21*, and *C1QBP*), are believed to participate in IBV infection by regulating innate immune response genes.

Screening of lncRNAs and their comparison between virus- and mock-infected cells revealed viral infection-associated host lncRNAs. In this study, we analyzed the expression patterns of lncRNAs in IBV-infected and mock-infected HD11 cells using RNA sequencing. The results showed 181 lncRNAs to be differentially expressed. Among these, 59 lncRNAs were up-regulated and 122 were down-regulated. Interestingly, we found that the majority of DE-lncRNAs, such as MSTRG.25416.43, MSTRG.6458.31, and MSTRG.24743.3, whose target genes (TARDBP, FMR1, and TRIM8) were mainly enriched in terms related to nucleic acid and protein metabolism, and not to terms related to immunity (although certain virus-related terms were also been enriched). The most likely reason could be that nucleic acids and proteins required for virus assembly in the cell are synthesized in large quantities during infection. Protein synthesis in cells is regulated by a variety of factors, including host lncRNAs. We speculated that IBV uses the host’s lncRNAs to produce viral nucleic acids and proteins required for replication. Moreover, early studies have demonstrated that the virus hijacks host-encoded lncRNAs to establish persistent infections [29]. Vesicular stomatitis virus, vaccinia virus, and herpes simplex virus 1 could hijack the lncRNA–ACOD1 complex to promote viral replication by activating the metabolic enzyme glutamic-oxaloacetic transaminase 2 [30, 31]. Furthermore, lncRNA NeST, Lethe, lncRNA-CMPK2, VIN, and NRON are used by the virus to facilitate infection or susceptibility [32].

The ceRNA hypothesis proposes that transcripts with shared microRNA (miRNA)-binding sites compete for post-transcriptional control. Recently, lncRNA HAND2-AS1 was found to work as the ceRNA of miR-3118 to suppress the proliferation and migration in breast cancer by upregulating *phlpp2* [33]. LncRNA-Six1 is a target of miR-1611 that functions as a ceRNA to regulate the expression of Six1 protein and fiber type switching in chicken myogenesis [34]. LncRNA MARL functioned as a ceRNA for miR-122 to control protein abundance of MAVS, thereby inhibiting the SCRV replication and promoting antiviral responses [35]. Previously, we demonstrated that gga-miR-30d inhibited IBV replication in HD11 cells by targeting USP47 [16]. MiR-146a-5p promoted IBV replication in HD11 cells by targeting IRAK2 and TNFRSF18 [17]. We further analyzed the changes in lncRNAs in IBV-infected HD11 cells. Based on gga-miR-30d and miR-146a-5p, we constructed the lncRNA-microRNA-mRNA ceRNA network that provided insights into the interplays in lncRNAs, microRNAs, and mRNAs. For example, lncRNA MSTRG.21445.2, identified as a lncRNA with a length of about 4000 bp in our study, was significantly up-regulated following IBV infection. It is located downstream of the *USP47* and overlaps with the pellino E3 ubiquitin protein ligase family member 2 (*PELI2*) gene on chicken chromosome 5. The function of *PELI2* in innate immunity has already been revealed [36, 37]. LncRNA MSTRG.21445.2 regulates IBV infection by competing with mRNAs of *USP47*, *IRAK2,* and *TNFRSF18* for gga-miR-30d and miR-146a-5p. Moreover, it may participate in natural immunity against IBV infection by acting on *PELI2* alone. Although these results provided new insights for studying the mechanism of IBV infection, bioinformatics-based predictions should be confirmed using wet-laboratory studies.

## Conclusion

We performed a comprehensive analysis of lncRNA and mRNA expression profiles in HD11 cells following IBV infection using RNA sequencing. We identified more than 2000 novel chicken lncRNAs. In addition, we identified several DE genes that may play pivotal roles in IBV infection using co-expression network analysis. We screened several DE lncRNAs related to IBV replication and cellular immunity, e.g., lncRNAs MSTRG.14220.1, MSTRG.21445.2, MSTRG.25416.43, MSTRG.6458.31, and MSTRG.24743.3. Further, we established a ceRNA network centered on gga-miR-30d and miR-146a-5p. We believe our results provide valuable insights into the mechanisms of IBV pathology.

## Methods

### Cell culture and virus infection

The HD11 cell line was kindly provided by Prof. Xin-An Jiao, Yangzhou University. The cells were cultured in Dulbecco’s modified Eagle’s medium (DMEM, Gemini, USA) supplemented with 10% fetal bovine serum (FBS, Gemini, USA), 100 IU/mL penicillin, and 100 μg/mL streptomycin sulfate (Solabio, USA). The IBV Beaudette strain (GenBank: DQ001339) was kindly gifted by Prof. Ding-Xiang Liu, Nanyang Technological University. The cells were infected with the IBV Beaudette strain (multiplicity of infection [MOI] = 2) and incubated in 5% CO_2_ at 37 °C for 1 h. Following the incubation, the cells were rinsed with phosphate-buffered saline (PBS, Gemini) and cultured at 37 °C in DMEM supplemented with 2% FBS (5% CO_2_). Controls included cells that were mock infected.

### RNA isolation and library construction

HD11 cells were divided into two groups with three biological replicates in each group: cells infected with IBV represented the experimental group (Exp 1, 2, and 3); mock-infected HD11 cells served as control (CK 1, 2, 3). Following the infection for 36 h, total RNA was prepared from these treatments by the TRIzol method (Invitrogen, USA) according to the manufacturer’s protocol. Total RNA quality was assessed on an Agilent 2100 Bioanalyzer (Agilent Technologies, Santa Clara, CA, USA). Sequencing libraries were generated using the rRNA-depleted RNA with NEBNext® Ultra™ Directional RNA Library Prep Kit for Illumina® (NEB, USA) following the manufacturer’s recommendations.

### Transcript sequencing and genome mapping

The libraries were paired-end sequenced (PE150, sequencing reads were 150 bp) at Personal Biotechnology Co., Ltd. (Shanghai, China) using the Illumina HiSeq 2000. The information on the quality of raw data in FASTQ format was calculated, following which the raw data was filtered using Cutadapt (v2.7) software. The clean data were obtained by removing the reads containing the adapter, reads containing poly-N, and low-quality reads. All subsequent analyses were based on high-quality clean data. The chicken genome GRCg6a (GenBank assembly accession: GCA_000002315.5) was used as the reference genome. The reference genome index was constructed by Bowtie2 (v2.4.1) and the filtered reads were mapped onto the reference genome using HISAT2 (2.1.0), the default mismatch was no more than two. The alignment region distribution of mapped reads was calculated.

### Coding potential and conserved analysis of lncRNAs

First, the raw data were filtered to obtain high-quality clean data. The HISAT2 (2.1.0) software was used to compare the filtered reads with the chicken genome as the reference. Based on the results of HISAT2 (2.1.0), StringTie (v1.2.4) software was used to assemble the transcripts. Transcripts with uncertain directions were removed and lncRNAs were screened in the remaining assembled transcript set using the following steps: (1) Transcripts with length ≥ 200 bp and the number of exons ≥2 were selected. (2) Only transcripts class-coded as “u” (unknown intergenic transcript), “i” (a transfrag falling entirely within a reference intron), and “x” (exonic overlap with reference on the opposite strand) were selected as candidate lncRNAs. (3) LncRNAs with cover > 3 in at least one sample, that is, those appearing at least thrice in the transcript of one sample, were screened. (4) To further obtain high-confidence lncRNAs, the coding potential of the candidate lncRNA was analyzed using PLEK (1.2), CNCI (v2) and PfamScan (1.6). (5) The mRNAs were compared with predicted lncRNAs in terms of transcript numbers, length, and exon numbers to describe the characteristics of lncRNAs.

### LncRNA target gene prediction

The target gene prediction of lncRNAs was divided into cis-target gene prediction and trans-target gene prediction. We screened for genes with protein-coding capabilities within 100 kb upstream and downstream of lncRNAs for cis-regulated target genes corresponding to lncRNAs. The target genes with trans-regulation were screened by calculating the expression correlation (Pearson’s correlation test) or co-expression analysis method between lncRNAs and mRNAs. We used the correlation coefficient > 0.95 and *p*-value < 0.05 to screen the trans-acting relationship between lncRNAs and mRNAs. Next, the lncRNA-mRNA co-expression network was constructed using Cytoscape.

### Identification and bioinformatics analyses of differentially expressed mRNAs and lncRNAs

StringTie (version 1.2.4) software was used to perform counts at the transcript level to obtain the original expression. Next, FPKM (fragments per kilobase of exon model per million fragments mapped) was used to normalize the expression. In general, FPKM > 1 demonstrated expression. DESeq was used to analyze the DEGs and DE lncRNAs in IBV-infected and control cells. The conditions for screening DEGs were differential expression multiples |log2FoldChange| > 1, significance *p*-value < 0.05.

Gene ontology (GO, http://geneontology.org/) and Kyoto encyclopedia of genes and genomes (KEGG, http://www.kegg.jp/) enrichment analyses were performed on DEGs and target genes of DE-lncRNAs. TopGO (2.40.0) was used to map all DEGs and target genes to each term in the GO database; the number of DEGs and target genes for each term was calculated, and the hypergeometric distribution was performed to calculate the DE lncRNA target genes. GO terms with corrected *p*-value ≤0.05 were considered significantly enriched. Similarly, KEGG automatic annotation server (KAAS) was used to perform pathway annotation using the entire genome as the background. The hypergeometric distribution was used to calculate significantly enriched pathways of DEGs and lncRNA target genes, and pathways with a *p*-value ≤0.05 were considered significantly enriched.

### Network analysis of miRNAs, lncRNAs, and genes

LncRNAs can directly bind to the target gene or interact with miRNA as a ceRNA (competitive endogenous RNA) to regulate the expression of the target gene. MiRanda (v3.3a) software was used to predict miRNA target genes. A putative competing endogenous RNA (ceRNA) network was imported to Cytoscape based on the screening of lncRNA-miRNA-mRNA pairs.

### Quantitative PCR and indirect immunofluorescence assay

Total RNA was isolated using the TRIzol reagent (Invitrogen, USA) and cDNA was synthesized using the PrimeScript RT reagent kit (TaKaRa). RT-qPCR was performed to define the relative mRNA expression of the genes using UltraSYBR Mixture (CWBio, Beijing, China) according to the manufacturer’s instructions. Relative fold changes in the gene expression were normalized against chGAPDH using the 2^-∆∆Ct^ threshold method. Indirect immunofluorescence assay (IFA) was used to observe the proliferation of the virus. Briefly, IBV-infected or mock-infected HD11 cells were incubated with mouse anti-IBV N protein monoclonal antibody (Novus Biologicals, USA) at 37 °C for 2 h. Next, the cells were incubated with a secondary antibody (Alexa Fluor 555-labeled donkey anti-mouse IgG (H + L), Beyotime) at 37 °C for 2 h, and stained with DAPI for 10 min before observing the cells under a fluorescence microscope.

### Statistical analysis

All data were analyzed using Origin 8.1. Multiple groups were compared by one-way analysis of variance (ANOVA) followed by Tukey’s multiple comparison tests. Data are expressed as mean ± standard deviation (SD) of three independent experiments. The *p*-values < 0.05 were considered significant.

## Supplementary Information

**Additional file 1.** Differentially expressed mRNA.

**Additional file 2.** Differentially expressed lncRNA.

**Additional file 3.** Cis-target lncRNA prediction.

**Additional file 4.** Trans-target lncRNA prediction.

**Additional file 5.** Immune related lncRNAs.

**Additional file 6.** lncRNA-miRNA-mRNA network.

## Data Availability

Raw RNA-seq data has been deposited into the National Center for Biotechnology Information (NCBI) under the BioProject accession PRJNA659912 (https://www.ncbi.nlm.nih.gov/search/all/?term=PRJNA659912/) and Sequence Read Archive (SRA) database under the accession SRR12543068, SRR12543069, SRR12543070, SRR12543071, SRR12543072, SRR12543073 (https://trace.ncbi.nlm.nih.gov/Traces/sra/?run=SRR12543068).

## Abbreviations

IBV: infectious bronchitis virus
IB: infectious bronchitis
lncRNAs: long non-coding RNAs
ceRNA: competing endogenous RNA
ncRNAs: non-coding RNAs
BP: biological process
CC: cellular component
MF: molecular function
IFA: indirect fluorescent immunoassay
GO: gene ontology
KEGG: Kyoto Encyclopedia of Genes and Genomes
DE: differentially expressed
PELI2: pellino E3 ubiquitin protein ligase family member 2
FPKM: fragments per kilobase of exon model per million reads mapped
